# Fetal MRI based brain atlas analysis detects initial in utero effects of prenatal alcohol exposure

**DOI:** 10.1093/cercor/bhad005

**Published:** 2023-02-17

**Authors:** Marlene Stuempflen, Ernst Schwartz, Mariana C Diogo, Sarah Glatter, Birgit Pfeiler, Patric Kienast, Athena Taymourtash, Victor U Schmidbauer, Lisa Bartha-Doering, Elisabeth Krampl-Bettelheim, Rainer Seidl, Georg Langs, Daniela Prayer, Gregor Kasprian

**Affiliations:** Department of Biomedical Imaging and Image-guided Therapy, Medical University of Vienna, Vienna 1090, Austria; Department of Biomedical Imaging and Image-guided Therapy, Medical University of Vienna, Vienna 1090, Austria; Department of Neuroradiology, Hospital Garcia de Orta, Almada 2805-267, Portugal; Department of Pediatrics and Adolescent Medicine, Medical University of Vienna, Vienna 1090, Austria; Department of Biomedical Imaging and Image-guided Therapy, Medical University of Vienna, Vienna 1090, Austria; Department of Biomedical Imaging and Image-guided Therapy, Medical University of Vienna, Vienna 1090, Austria; Department of Biomedical Imaging and Image-guided Therapy, Medical University of Vienna, Vienna 1090, Austria; Department of Biomedical Imaging and Image-guided Therapy, Medical University of Vienna, Vienna 1090, Austria; Department of Pediatrics and Adolescent Medicine, Medical University of Vienna, Vienna 1090, Austria; Department of Obstetrics and Feto-maternal Medicine, Medical University of Vienna, Vienna 1090, Austria; Department of Pediatrics and Adolescent Medicine, Medical University of Vienna, Vienna 1090, Austria; Computational Imaging Research Lab, Department of Biomedical Imaging and Image-Guided Therapy, Medical Univers of Vienna, Vienna 1090, Austria; Department of Biomedical Imaging and Image-guided Therapy, Medical University of Vienna, Vienna 1090, Austria; Department of Biomedical Imaging and Image-guided Therapy, Medical University of Vienna, Vienna 1090, Austria

**Keywords:** brain atlas, fetal alcohol spectrum disorders, fetal brain, magnetic resonance imaging, prenatal alcohol exposure

## Abstract

Prenatal alcohol exposure (PAE) can change the normal trajectory of human fetal brain development and may lead to long-lasting neurodevelopmental changes in the form of fetal alcohol spectrum disorders. Currently, early prenatal patterns of alcohol-related central nervous system changes are unclear and it is unknown if small amounts of PAE may result in early detectable brain anomalies.

This super-resolution fetal magnetic resonance imaging (MRI) study aimed to identify regional effects of PAE on human brain structure. Fetuses were prospectively assessed using atlas-based semi-automated 3-dimensional tissue segmentation based on 1.5 T and 3 T fetal brain MRI examinations. After expectant mothers completed anonymized PRAMS and TACE questionnaires for PAE, fetuses without gross macroscopic brain abnormalities were identified and analyzed. Linear mixed-effects modeling of regional brain volumes was conducted and multiple comparisons were corrected using the Benjamini–Hochberg procedure. In total, 500 pregnant women were recruited with 51 reporting gestational alcohol consumption. After excluding confounding comorbidities, 24 fetuses (26 observations) were identified with PAE and 52 age-matched controls without PAE were analyzed. Patients with PAE showed significantly larger volumes of the corpus callosum (*P* ≤ 0.001) and smaller volumes of the periventricular zone (*P* = 0.001). Even minor (1–3 standard drinks per week) PAE changed the neurodevelopmental trajectory.

## Introduction

Central nervous system (CNS) development is a complex process starting within the first weeks of gestation (Elshazzly et al. 2021). It is highly susceptible to external factors such as teratogenic substances, which may affect organogenesis including the CNS structures (Elshazzly et al. 2021). Teratogenic properties of prenatal alcohol exposure (PAE) have been proven to have the ability to alter the development of various organ systems (Caputo et al. 2016) potentially resulting in a wide spectrum of physical and psychological long-term effects. Affected children may suffer from a variety of disease manifestations, which are commonly summarized as fetal alcohol spectrum disorders (FASD), among which fetal alcohol syndrome (FAS) is considered to be the most severe (Gupta et al. 2016).

A recent meta-analysis by Popova et al. determined the prevalence of alcohol use during pregnancy to be 9.8% and the prevalence of FAS in the general population 14.6 per 10,000 people (Popova et al. 2017). The meta-analysis by Lange et al. (2017) determined the prevalence of FASD as 8/1,000 in the general population and 1/13 pregnant women, who consumed alcohol during gestation, to deliver a child with FASD. Despite the epidemiologic significance of this condition, maternal alcohol consumption during pregnancy is underreported and frequently remains undetected (Lange et al. 2017).

Various studies have investigated the long-term effects of PAE on neurodevelopment in humans postnatally using magnetic resonance imaging (MRI) and thereby identified several associated cerebral anomalies: among them reduction of overall brain volume as well as malformations of the corpus callosum (CC; Lebel et al. 2011). Still, there is a lack of data concerning the effect of PAE on CNS structures during the early prenatal period and on human CNS development in general. Although it is widely known that CNS structures are vulnerable to the effects of PAE, very few studies have examined developmental trajectories of these structures in utero. Volumetric analysis of postmortem fetal MRI has been shown to provide a detailed analysis of fetal brain compartments in unexposed fetuses with correlation of histological sections (Vasung et al. 2020). The goal of this study was to identify in-vivo changes in fetal MRI requiring more detailed measuring techniques compared with standard 2-dimensional volumetry currently used in clinical practice. Thus, we applied advanced computerized postprocessing (“MR super-resolution”) to perform a high-resolution atlas-based analysis of fetal brain MRI data (Ebner et al. 2020). Neuroimaging based fetal brain atlases can be regarded as quantitative “maps,” which allow to detect, localize, and objectify transient deviations from normal fetal brain development. This opens new possibilities in the detection and characterization of the early effects of prenatal exposure to toxic substances—such as alcohol.

The aim of this prospective, atlas-based fetal case-control MRI study was to detect regional effects of maternal alcohol consumption on fetal neurodevelopment and its quantification using volumetric measurements. We hypothesized that there is a selective vulnerability of specific compartments of the fetal brain. The investigated substructures of the fetal brain included the cortex, subcortical parenchyma (including the subcortical layers between but not including the cortical plate and subventricular), periventricular zone (PZ; including the ventricular and subventricular zones), ganglionic eminence (GE), ventricular system, CC, deep gray nuclei (basal ganglia and thalamus), brainstem, cerebellum, external cerebrospinal fluid (CSF) spaces, and hippocampi bilaterally. Selection of these regions was based on previously published literature of postnatal and histological studies investigating PAE exposure during pregnancy (Norman et al. 2009; Lebel et al. 2011; Donald et al. 2015; Jacobson et al. 2017; Warton et al. 2018). In addition to raising awareness for the socio-economic importance of prevention, detection, and support of families affected by this frequently undiagnosed condition, the results of this study can serve as proof of principle that the effects of early exposure to toxins can be detected and quantified by atlas-based analysis of fetal brain imaging data.

## Materials and methods

### Subjects

Women with singleton pregnancies undergoing fetal MRI at a tertiary care center were prospectively recruited from November 2018 until August 2021. This study was approved by the institutional ethics board in accordance with the Helsinki Declaration of 2013 (World Medical Association 2013). All examinations were clinically indicated and referred by prenatal ultrasound centers and informed consent was obtained prior to MRI. Maternal medical history, gestational ages as determined by ultrasound (given in gestational weeks (GE) and days post menstruationem), and alcohol consumption prior to and during gestation were identified utilizing 2 standardized questionaries Pregnancy Risk Assessment Monitoring System (PRAMS) (Shulman et al. 2018) and Tolerance, Annoyance, Cutting-down, Eye-opener (TACE) (Sokol et al. 1989) with a reportedly high sensitivity for detecting alcohol intake during pregnancy.

**Fig. 1:**
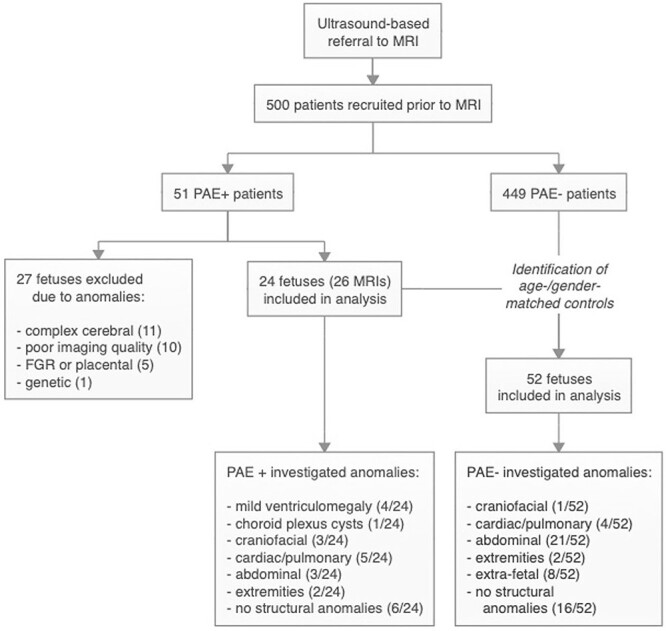
Flowchart depicting the patient recruitment and selection process. PAE+: alcohol exposed group, PAE−: nonexposed group.

After informed consent was obtained and imaging acquisition was concluded, inclusion and exclusion criteria were applied: Fetuses were included in further analysis if the gestational age was determined by first-trimester ultrasound, ultrasound organ screening had been conducted, high-quality super-resolution reconstruction was available, and an absence of severe structural cerebral (e.g. complex combinations of structural defects involving multiple areas of the fetal brain, dysraphism, and hemorrhage), severe cardiac (e.g. hypoplastic left heart), severe body anomalies (e.g. omphalocele and body stalk malformation), and maternal drug abuse was determined by MRI or ultrasound and genetic testing. Patients with the aforementioned anomalies were excluded as their presence would act as confounding comorbities. Participants were assigned to the group with (PAE+) or the control group without (PAE−). Participants exposed to any reported amount of alcohol greater than zero were allocated to the PAE+ group. Only cases with no alcohol exposure throughout the entire pregnancy were allocated to the PAE− group. For statistical analysis, PAE+ participants were paired with age-matched PAE− control cases in a 1:2 (PAE+ : PAE−) ratio (Fig. 1). Missing data were attempted to be completed using institutional patient records. Cases with incomplete data were excluded from further analysis.

### Fetal MRI

Fetal MRI scans were conducted using 1.5 T (Philips Ingenia/Intera, Best, Netherlands) and 3 T magnets (Philips Achieva, Best, Netherlands). The mother was examined in a supine or, if necessary, left recumbent position to achieve sufficient imaging quality and a body coil was used. The examinations were performed within 45 min and neither sedation nor MRI contrast medium were applied. MRI scans were done in accordance with the 2017 International Society of Ultrasound in Obstetrics & Gynecology Practice Guidelines (Prayer et al. 2017) and both the fetal head and body were imaged. Fetal brain imaging included T2-weighted sequences in 3 orthogonal planes (slice thickness 2.0–4.5 mm, echo time = 100–140 ms, field of view = 200–230 mm, and in-plane resolution 0.62/0.62–1.17/1.17 mm) of the fetal head.

### Postprocessing

Postprocessing was conducted in a similar methodology as done by Gholipour et al. (2017) and Schwartz et al. (2021). For each examination, at least 3 acquisitions of T2-weighted sequences of the fetal brain were obtained in 3 orthogonal planes based on fetal bodily organs. In postprocessing, imaging data were denoised (Coupe et al. 2008), in-plane super-resolution was generated (Dong et al. 2016), and automatic brain masking was conducted (Ebner et al. 2020). The resulting data were comprised of a single 0.5-mm isotropic volume using combined slice-wise motion correction and a volumetric super-resolution algorithm (Ebner et al. 2020). Resulting volumes were subsequently aligned to a common reference space (Gholipour et al. 2017).

**Fig. 2:**
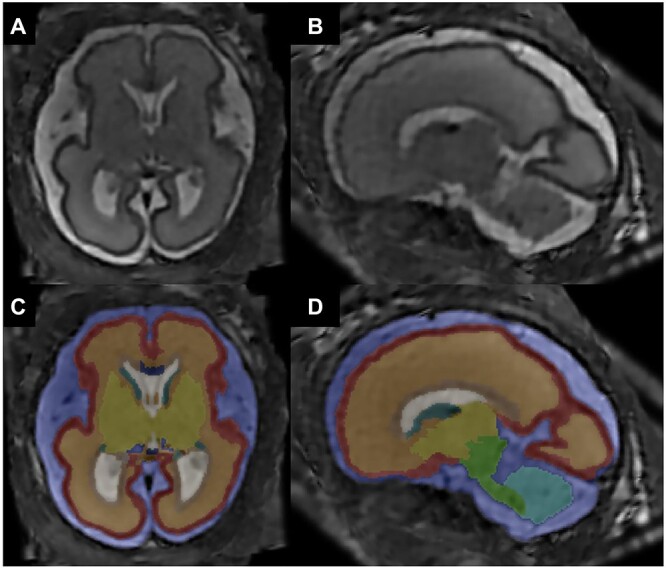
MRI super-resolution reconstruction and atlas-based tissue segmentation. **A, B**) Postprocessed T2-weighted MRI super-resolution reconstructions in axial (left) and sagittal (right) planes of a fetus at 26+6 gestational weeks (GW). **C, D)** Respective manually corrected atlas-based tissue segmentation. Color coding: blue—external CSF-spaces, red—cortex, orange—subcortical parenchyma (including the subcortical layers between, but not including, the cortical plate and subventricular), brown—periventricular zone (including the ventricular and the subventricular zone), dark green—ganglionic eminence, white—ventricular system, dark blue—corpus callosum, yellow—deep gray nuclei (basal ganglia and thalamus), light green—brainstem, light blue—cerebellum, turquoise—left hippocampus, and gray—right hippocampus.

Segmentation of cerebral regions of interest was performed by nonrigid mapping of a publicly-available, spatiotemporal, and anatomical fetal brain atlas for each case (Gholipour et al. 2017). These included the cortex, subcortical parenchyma (including the subcortical layers between, but not including, the cortical plate and subventricular), PZ (including the ventricular and the subventricular zone), GE, ventricular system, CC, deep gray nuclei (basal ganglia and thalamus), brainstem, cerebellum, external cerebrospinal fluid (CSF) spaces, and hippocampi bilaterally. To account for inaccuracies in the ultrasound-based estimation of exact conception dates as well as individual variability in neuronal development, atlases covering prior and consecutive weeks of estimated gestational age for each case were also included and merged using a label fusion technique (Wang et al. 2013). The resulting super-resolution data and tissue segmentation were scored by 2 raters (MS—in-training and GK—neuroradiologist) independently using a 5-point image quality scale. Cases that did not meet high-quality standards (score > 2) were excluded from the analysis. Cases of sufficient super-resolution quality were manually corrected by 3 raters (MS, PK—both in-training, and MCD—neuroradiologist) using the open-source application ITK-SNAP (Yushkevich et al. 2006; Fig. 2). The manually corrected segmentations were furthermore visually inspected by another neuroradiologist (GK) to allow for the highest level of accuracy. Discrepancies were corrected based on a consensus of the neuroradiologists. Segmentations were performed in anatomical correlation with the revised classification of the Boulder Committee with one adaptation (Bystron et al. 2008): To achieve the highest level of accuracy, the subventricular and the ventricular zone were combined and described as PZ. Similarly, histological fetal atlantes by Bayer and Altman were used as a reference guide to define the GE (Bayer and Altman 2004, 2005). Regarding the CC, delineation of its lateral borders can be difficult (Gholipour et al. 2017). To account for this, we defined the lateral borders of the CC, which can be identified by having a hypointense signal on T2-weighted super-resolution reconstructions, with consideration of the adjacent structures including the interhemispheric fissure/interhemispheric cortical structures, the lateral aspects of the normal-sized lateral ventricles and the deep gray nuclei while. In our patient collective, we observed the CC to be of a more hypointense signal in many cases compared with the PZ, which could however not be used as the exclusive approach for differentiation. Thus, a combination of signal intensity and spatial relationship to the surrounding landmarks was used to attempt an accurate denomination of the lateral callosal border. Volumetric data were extracted and calculations for all cerebral regions were made throughout the investigated gestational ages.

### Statistical analysis

Linear mixed-effects modeling of the volume of investigated structures of the fetal head was performed with fixed effects for gestational age and PAE-status. As 2 patients were scanned twice at different gestational ages, a random effect for patient identity was added. Individual models for all structures were built. From each, the significance of the effect of PAE-status was further corrected for multiple comparisons applying the Benjamini–Hochberg procedure. *P*-values used for significance were defined as *P* < 0.05. Compartment-based mean Hausdorff distance and Sørensen–Dice-coefficient were calculated to verify the accuracy of the manually corrected segmentations for a test-set of 15 patients.

All calculations were performed using R version 4.0.3. Mixed-effects modeling was performed using LME4.

## Results

### Study population

In total, 500 women were initially recruited in this study (Table 1 and Fig. 1). Mean gestational age at the time of MRI examination was 27.61 GW (standard deviation, SD 3.94) and 27.57 GW (SD 3.94) for the PAE+ and PAE− groups, respectively, and ranged from 21 to 37 GW (Table 1). Gestational ages among PAE+ and their respectively matched PAE− cases varied with a maximum age difference of 4 gestational days. The mean maternal age for the PAE+ and PAE− groups were 30.8 (SD 6.14) and 31.1 years (SD 4.94), respectively. In the PAE+ group 33.3% of fetuses were females vs. 34.6% in the PAE− group. A table indicating the reasons for referral to fetal MRI as well as final diagnoses is provided as Supplementary Material.

**Table 1:** Demographics sample size given in number of fetuses (number of scans).

**Participants**	**PAE+**	**PAE−**
**Sample size**	24 (26)	52 (52)
**Gestational age (GW)**	27.61 (3.94)	27.57 (3.94)
**Maternal age (years)**	30.8 (6.14)	31.1 (4.94)
**Fetal sex (female)**	33.3%	34.6%

Ages given in mean (standard deviation). PAE+: alcohol exposed group, PAE**−**: nonexposed group, GW: gestational weeks.

On average, the patients in the PAE+ group consumed one to 3 standard drinks per week and admitted to at least one binge-drinking episode of 4 or more drinks on one occasion during pregnancy. According to the US National Institute on Alcohol Abuse and Alcoholism (NIAAA), one standard drink is defined to contain roughly 14 g of pure alcohol (NIAAA, 2021). Among the PAE− group, fetuses were exposed to neither regular alcohol consumption nor to singular binge-drinking events. Evaluation of precise timing of alcohol consumption was not possible due to a high level of recall uncertainty among patients.

Overall, 51 fetuses with (PAE+) and 449 without (PAE−) prenatal alcohol exposure were identified (Fig. 1). After fetal MRI examination, 27 PAE+ fetuses had to be excluded for the following reasons: additional complex cerebral anomalies (11), fetal growth restriction (FGR)/placental anomalies (5), confirmed genetic anomalies (trisomy 13, 1), or poor postprocessing image resolution (10). Twenty-four PAE+ fetuses (undergoing a total of 26 MRI scans) were finally included in the analyses. In addition, 52 age-matched PAE− fetuses (undergoing a total of 52 MRI scans) without brain anomalies were selected for the PAE− control group to achieve a 1:2 matching ratio.

### Investigated anomalies

For the PAE+ group, MRI scans detected mild ventriculomegaly (4/24), choroid plexus cysts (1/24), craniofacial deformities without the involvement of the brain (3/24), cardiac/pulmonary defects (pulmonary sequestration, right-sided aortic arch, and aortic isthmus stenosis) (5/24), abdominal abnormalities (3/24), and abnormal extremities (2/24). No structural anomalies were identified in the remaining 6/24 fetuses.

Among the fetuses of the PAE− group, craniofacial deformities without the involvement of the brain (1/52), cardiac/pulmonary defects (4/52), abdominal abnormalities (21/52), abnormal extremities (2/52), and extra-fetal anomalies (8/52) were detected. 16/52 PAE− fetuses were categorized to be normal.

### Volumetric results

**Fig. 3:**
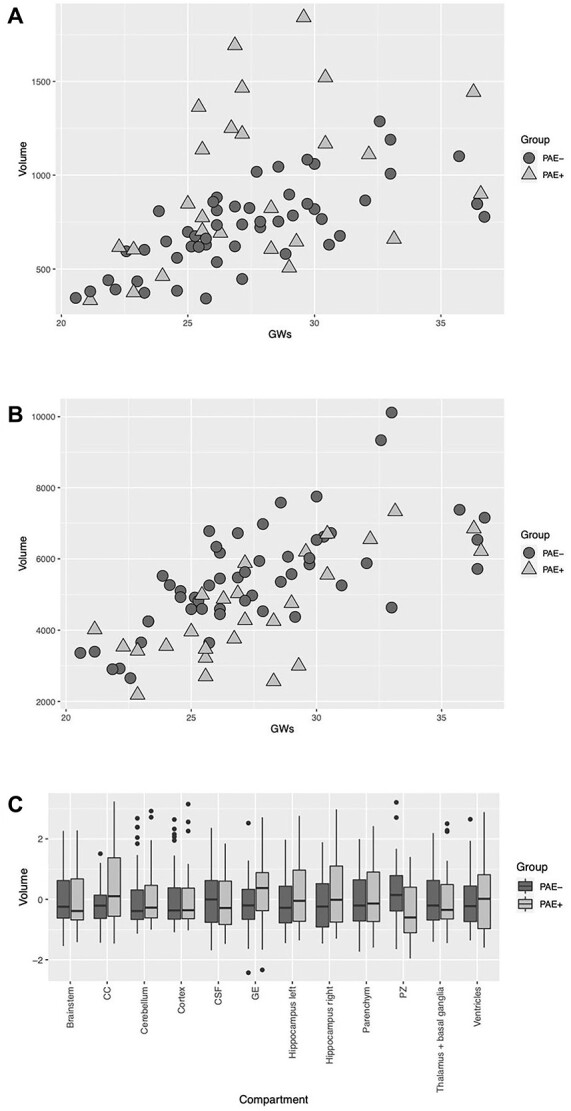
Distribution of regional brain volumes with and without prenatal alcohol exposure (PAE) throughout gestation. **A**) Volumes of the corpus callosum given in mm^3^ and gestational weeks (GW). **B**) Volumes of the periventricular zone given in mm^3^ and gestational weeks (GW). **C**) Volumes of brainstem, corpus callosum (CC), cerebellum, cortex, external cerebrospinal fluid (CSF) spaces, ganglionic eminence (GE), left and right hippocampus, subcortical parenchyma, periventricular zone (PZ), deep gray nuclei (thalamus and basal ganglia), and ventricular system throughout gestation. Color coding: light gray/triangles—PAE+ group and dark gray/circles—PAE− group.

**Fig. 4:**
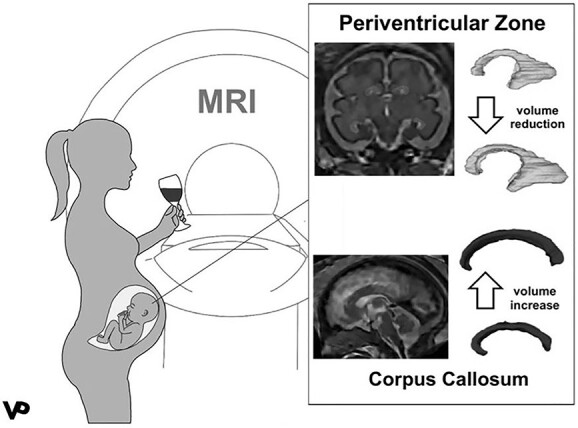
Effects of prenatal alcohol exposure (PAE). PAE leads to a volume reduction in the periventricular zone and a volume increase in the corpus callosum. Color coding: light gray — periventricular zone, dark gray — corpus callosum.

**Fig. 5:**
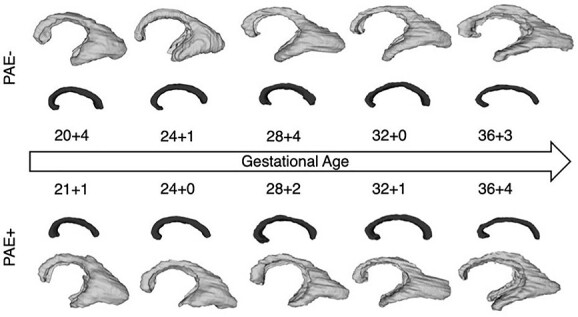
Longitudinal growth trajectories of the periventricular zone and the corpus callosum in fetuses with (PAE+) and without (PAE−) prenatal alcohol exposure. The volumetric relations of included images are not representative: The scaling is adjusted to allow for an easy comparison of shape and structure of the respective compartments. Gestational ages given in gestational weeks + days. Color coding: light gray — periventricular zone, dark gray — corpus callosum.

Linear mixed-effect models of the volume of each segmented structure revealed a significant effect for PAE-status on the CC volume (*P* < 0.001, 0.95 confidence interval, CI [99.29–346.45]; Fig. 3 and Table 2) and on the volume of the PZ (*P* = 0.001, 0.95 CI [−1408.05 to −381.57]; Fig. 3 and Table 2) that survived multiple comparison correction at *q* = 0.006 each. Thus, a statistically significant increase in volume of the CC and a decrease in volume of the PZ were identified (Figs. 4 and 5). Models for the remaining structures—including cortex, subcortical parenchyma, GE, ventricular system, deep gray nuclei (basal ganglia and thalamus), brainstem, cerebellum, external CSF spaces, and bilateral hippocampi as well as total brain volume—did not show a significant effect of the PAE-status on the volumes (Table 3). Group results of volumes in the 12 regional brain compartments are presented in Fig. 3.

**Table 2:** Effect of PAE-status on corpus callosum and periventricular zone volume.

	**Corpus callosum**			**Periventricular zone**		
**Predictors**	**Estimates**	**Confidence interval**	***P*-value**	**Estimates**	**Confidence interval**	***P*-value**
**(Intercept)**	−374.56	−793.15 to 44.04	79	−1,695.38	−3,366.97 to −23.80	47
**Gestational age**	5.71	3.58–7.85	**<0.001**	37.22	28.69–45.75	**<0.001**
**PAE-status**	222.87	99.29–346.45	**<0.001**	−894.81	−1,408.05 to −381.57	1
**Random effects**						
**σ** ^**2**^	67,103.44			456,916.57		
**τ**_**00 Patient**_	1,640.57			684,726.75		
**Intraclass correlation coefficient**	0.02			0.60		
***n***_**patients**_	76			76		
***n***_**observations**_	78			78		
**Marginal *R*** ^**2**^ **/conditional *R*** ^**2**^	0.343 / 0.358			0.516 / 0.806		

Bold results highlight *P*-values of < 0.05, deemed statistically significant.

Regarding the accuracy of segmentation, mean Hausdorff distances and Sørensen–Dice-coefficients were calculated for a test-set of 15 patients (Table 4). The mean Hausdorff distances among the segmentations of both raters ranged from 0.019 to 0.525, whereas the Sørensen–Dice-coefficients ranged from 0.628 to 0.995. These variations in accuracy between both raters were most likely due to the great difference in the volumetric size of the segmented structures: Smaller structures lead to lower Sørensen–Dice-coefficient-values caused by even minor segmentation discrepancies between raters. Overall mean value of the Sørensen–Dice-coefficient for all segmented regions was 0.977, indicating a very high level of correlation among the analyzed segmentations.

## Discussion

This is the first compartmental, volumetric atlas-based analysis of the effects of PAE on fetal brain development using segmentation-based super-resolution MRI. This approach of advanced postprocessing of fetal MRI data provides initial insights into the selective vulnerability of specific fetal brain structures—even in cases with normal diagnostic fetal brain imaging results. PAE was associated with an increased volume of the CC and a volume reduction of the PZ during the mid-second and third trimesters of pregnancy. Regional brain volumes of transient brain structures such as the PZ as well as the dynamically changing CC were found to be altered despite a relatively low amount of maternal alcohol consumption (mean = 1–3 drinks/week) in the exposed group.

The presented findings help to explain the diversity of structural brain alterations found by postnatal neuroimaging studies (Norman et al. 2009; Lebel et al. 2011; Roediger et al. 2021): Fetal brain compartments associated with neuronal proliferation and differentiation (the PZ) as well as structures linked to ongoing axonal growth, selection, targeting, and guidance (the CC) are most severely affected by PAE and thus lead to global and diffuse changes of brain structure and function at later stages of life.

The observed changes in fetal development are caused by a highly complex set of intercellular, intracellular, and epigenetic factors (Chung et al. 2021): It is hypothesized that various mechanisms play a role at different timepoints during gestation affecting different components and steps of fetal development. To specify just one affected mechanism, intercellular connectivity can be altered following PAE following a change in the cell adhesion molecule L1, which prevents neurons from clumping together and establishing normal cell-to-cell contact required for physiological growth and development (Miller and Robertson 1993; Ramanathan et al. 1996; Goodlett and Horn 2001).

According to the developmental and genetic classification for malformations of cortical development by Barkovich et al. (2012), microcephaly is a disorder of neuronal proliferation and a severe phenotypic feature of FAS most strikingly affecting the PZ. The presented volume reductions of the PZ can be explained by neurobiological changes on a cellular level and confirm the existence of profound negative effects of early alcohol exposure on the proliferative zones of the human brain. Within the PZ, a variety of neuronal progenitor cells give rise to the excitatory and inhibitory interneurons of the future and forming cortex (Shohayeb et al. 2021). Furthermore, alcohol negatively impacts neuronal migration: Radial glial cells (RGCs) are unspecialized cells, which can give rise to both neurons and astrocytes (Götz et al. 2002) and constitute an important part of the radial glial scaffolding guiding neuronal migration (Nowakowski et al. 2016). Alcohol-induced alterations of radial glial cell development thus affect later cortical development (Miller and Robertson 1993). Moreover, radial glial fiber formation is altered by PAE and causes abnormal differentiation of glial fibrillary acidic protein δ-positive RGCs into astrocytes rather than into neurons (Li et al. 2021). This not only causes an abnormal migration pattern of neuronal cells but also a potential imbalance in the cellular composition of proliferative brain structures.

Our data suggest a possibly transient increase in the size of the CC associated with PAE as previously published data described reduced volumes in the postnatal period (Norman et al. 2009; Lebel et al. 2011; Donald et al. 2015; Jacobson et al. 2017; Warton et al. 2018). There is evidence for the presence of exuberant axonal projections along the corticospinal tracts as well as the CC (Stanfield 1992; Luo and O’Leary 2005). Innocenti reported a loss of a large proportion of these projections between birth and adulthood in cats (Innocenti 1981). Similarly, human midgestational fetuses have twice as many callosal axons as term neonates, whereas 2-year-old infants have even fewer (O’Leary et al. 1981; Sarnat 2008). This is the result of axonal selection and the later occurring synaptic reduction described as synaptic pruning (Innocenti 1981). So far, the phenomenon of pruning of exuberant callosal fibers is poorly understood in humans.

The result of increased callosal size may be interpreted by following the concept that PAE results in a delay in synaptic pruning, abnormal axon guidance, and subsequently altered neuronal connectivity (Mathews et al. 2021), detectable as early structural and volumetric alterations in the trajectory of callosal growth.

An alternative explanation could be provided by LaMantia and Rakic (1990), who conducted histological studies of the CC in rhesus monkeys and hypothesized that this massive reduction in axonal numbers might be caused by the axons varying origins, whereas many of the axons eliminated belong to the earlier generated neurons situated in the infragranular cortical layers, they suspected a larger proportion of later-generated callosal neurons, which settle in the supragranular layers II and III, to survive or retain interhemispheric axons (LaMantia and Rakic 1990). Transient callosal septa have also been shown to have a crucial role in axon guidance, resulting in physiological changes in callosal thickness and shape (Culjat and Milošević 2019). Thus, another possible explanation for the observed imaging findings might be a shift in the spatiotemporal development and migration pattern of these axons.

The window of highest vulnerability of the human CNS following PAE is thought to be during the third and fourth gestational week, which is much earlier than the first appearance of the CC (Rakic and Yakovlev 1968), highlighting the potential of PAE to affect developmental dynamics and resulting in a shift in developmental trajectories. However, our investigated patient collective did not yield a sufficient sample size to calculate a timepoint of highest vulnerability towards PAE. Larger patient cohorts of alcohol exposed fetuses would be required to sensitively correlate the timing of PAE to the severity of identified anomalies.

**Table 3:** Overview of effect sizes and *P*-values for gestational age and PAE-status on all investigated brain compartments.

**Compartment**	***P*-value for gestational age**	***P*-value for PAE-status**	**Effect size for gestational age**	**Effect size for PAE-status**
**Cortex**	<0.001	0.96	**−**3,054.167	462.607
**Brainstem**	<0.001	0.84	1,046.348	1.193.413
**Subcortical parenchyma**	<0.001	0.84	51.021	**−**57.922
**Deep gray nuclei**	<0.001	0.58	827.842	2,117.849
**Cerebellum**	<0.001	0.96	115.013	16.632
**Ventricular system**	<0.001	0.42	37.912	519.360
**Periventricular zone**	<0.001	0.006	37.221	**−**894.810
**Hippocampus right**	<0.001	0.18	7.692	65.820
**Hippocampus left**	<0.001	0.18	7.939	52.461
**Corpus callosum**	<0.001	0.006	5.713	222.869
**Ganglionic eminence**	0.77	0.18	**−**0.250	88.888

**Table 4:** Interrater-correlation—overview of compartment-based Hausdorff distance and Sørensen–Dice-coefficient values for a test-set of 15 patients segmented by 2 raters independently.

**Compartment**	**Mean Sørensen–Dice-coefficient**	**Mean Hausdorff distance**
**External CSF spaces**	0.958	0.070
**Cortex**	0.995	0.019
**Brainstem**	0.949	0.178
**Subcortical parenchyma**	0.989	0.040
**Deep gray nuclei**	0.969	0.124
**Cerebellum**	0.888	0.196
**Ventricular system**	0.975	0.055
**Periventricular zone**	0.638	0.374
**Hippocampus right**	0.765	0.357
**Hippocampus left**	0.760	0.412
**Corpus callosum**	0.754	0.289
**Ganglionic eminence**	0.628	0.525
**Overall**	0.856	0.220

### Clinical implications

In light of these results, thorough and standardized screening for maternal alcohol consumption should be once again emphasized and must be conducted for all pregnant patients throughout their pregnancy, as their drinking behavior might change during the course of their gestation. MRI is an important tool to detect and differentiate associated anomalies at early gestational dates, allowing for the initiation of specialized treatment and support of families affected by FASD, which are frequently undiagnosed and thus often an invisible burden.

Furthermore, quantification of the callosal thickness using 2-dimensional measurements in ultrasound and MRI yields abnormal values only in cases of extreme callosal enlargement and is thus not a reliable measurement approach (Achiron and Achiron 2001; Shinar et al. 2016). With regards to the PZ, data of 2-dimensional size measurements have not been investigated. Three-dimensional super-resolution-based MRI volumetry provides a novel and promising technique to reliably assess and measure these brain structures.

### Research implications

The results of this study show that even low amounts of PAE leads to a distortion of normal brain maturation and a subsequently abnormal developmental trajectory resulting in the observed changes in fetal MRI. This emphasizes the role of MRI not only in the investigation of congenital malformations but also in the presence of epigenetic factors such as PAE. Larger studies are required to confirm and refine our findings and to study whether there is a correlation between the level of PAE and the severity of associated anomalies.

### Strengths and limitations

This study is the first compartmental, volumetric atlas-based analysis of the effects of PAE on fetal brain development using segmentation-based super-resolution MRI. We were able to identify brain regions most vulnerable to alcohol exposure during fetal neurodevelopment—the detrimental time-period responsible for the lifelong effects of PAE.

Because of the nature of this study, the precise mechanism of alcohol-induced changes in the fetal brain cannot be determined. Furthermore, we acknowledge a lack of histological correlation with MRI imaging of our subjects. However, manual segmentation was done in accordance with the histological fetal atlantes by Bayer and Altman (2004, 2005) and the revised classification of the Boulder Committee (Bystron et al. 2008).

We acknowledge the potential risk of underreporting alcohol consumption within our patient collective. However, the applied TACE questionnaire confidently enables physicians to predict postnatal neurodevelopmental outcomes (Chiodo et al. 2010) and thus serves as reliable tool in the detection of PAE. FASD is associated with a variety of CNS and body malformations (Norman et al. 2009; Lebel et al. 2011; Roediger et al. 2021), for this reason we included fetuses with mild extra-CNS abnormalities. Even if impairment of brain growth has been documented in cases with cardiac defects, no involvement of the CC and the PZ have been reported so far.

Lateral borders of the CC can be difficult to delineate. Furthermore, previous histological studies have shown that the border between the CC and the PZ is partially ill-defined (Žunić Išasegi et al. 2018): This is due to the fact, that the PZ does also include some portions of the callosal fiber system. Even at early mid-gestation not all commissural axons have completely reached the CC (Richards et al. 2004)—an intrinsic limitation to imaging based delineation. However, we implemented the abovementioned considerations to ensure a highly accurate separation based on fetal MR images alone. This represents the clinical practice, where histological correlation is not available for every investigated patient.

Lastly, correlation of alcohol dosage with severity of MRI findings was not possible because of the limited sample size.

## Supplementary Material

Supplementary_material_bhad005

## Data Availability

Data generated or analyzed during this study are available from the corresponding author upon reasonable request.

## References

[ref1] Achiron R, Achiron A. Development of the human fetal corpus callosum: a high-resolution, cross-sectional sonographic study. Ultrasound Obstet Gynecol. 2001:18(4):343–347. 10.1046/j.0960-7692.2001.00512.x. 11778993

[ref2] Barkovich AJ, Guerrini R, Kuzniecky RI, Jackson GD, Dobyns WB. A developmental and genetic classification for malformations of cortical development: update 2012. Brain. 2012:135(Pt 5):1348–1369. 10.1093/brain/aws019. 22427329 PMC3338922

[ref3] Bayer SA, Altman J. Atlas of human central nervous system development. In: The human brain during the third trimester. Boca Raton, FL 33487-2742: CRC Press, Taylor & Francis Group, LLC; 2004

[ref4] Bayer SA, Altman J. Atlas of human central nervous system development. In: The human brain during the second trimester. Boca Raton, FL 33487-2742: CRC Press, Taylor & Francis Group, LLC; 2005

[ref5] Bystron I, Blakemore C, Rakic P. Development of the human cerebral cortex: Boulder Committee revisited. Nat Rev Neurosci. 2008:9(2):110–122. 10.1038/nrn2252. 18209730

[ref6] Caputo C, Wood E, Jabbour L. Impact of fetal alcohol exposure on body systems: a systematic review. Birth Defects Res C Embryo Today. 2016:108(2):174–180. 10.1002/bdrc.21129. 27297122

[ref7] Chiodo LM, Sokol RJ, Delaney-Black V, Janisse J, Hannigan JH. Validity of the T-ACE in pregnancy in predicting child outcome and risk drinking. Alcohol. 2010:44(7–8):595–603. 10.1016/j.alcohol.2009.08.009. 20053522 PMC2891940

[ref8] Chung DD, Pinson MR, Bhenderu LS, Lai MS, Patel RA, Miranda RC. Toxic and teratogenic effects of prenatal alcohol exposure on fetal development, adolescence, and adulthood. Int J Mol Sci. 2021:22(16):8785. 10.3390/ijms22168785. 34445488 PMC8395909

[ref9] Coupe P, Yger P, Prima S, Hellier P, Kervrann C, Barillot C. An optimized blockwise nonlocal means denoising filter for 3-D magnetic resonance images. IEEE Trans Med Imaging. 2008:27(4):425–441. 10.1109/TMI.2007.906087. 18390341 PMC2881565

[ref10] Culjat M, Milošević NJ. Callosal septa express guidance cues and are paramedian guideposts for human corpus callosum development. J Anat. 2019:235(3):670–686. 10.1111/joa.13011. 31070791 PMC6704273

[ref11] Donald KA, Eastman E, Howells FM, Adnams C, Riley EP, Woods RP, Narr KL, Stein DJ. Neuroimaging effects of prenatal alcohol exposure on the developing human brain: a magnetic resonance imaging review. Acta Neuropsychiatr. 2015:27(5):251–269. 10.1017/neu.2015.12. 25780875

[ref12] Dong C, Loy CC, Tang X. Accelerating the super-resolution convolutional neural network. In: Leibe B, Matas J, Sebe N, Welling M, editors. Computer vision – ECCV 2016. Cham: Springer International Publishing (Lecture Notes in Computer Science).; 2016. pp. 391–407

[ref13] Ebner M, Wang G, Li W, Aertsen M, Patel PA, Aughwane R, Melbourne A, Doel T, Dymarkowski S, De Coppi P et al. An automated framework for localization, segmentation and super-resolution reconstruction of fetal brain MRI. NeuroImage. 2020:206:116324. 10.1016/j.neuroimage.2019.116324. 31704293 PMC7103783

[ref14] Elshazzly M, Lopez MJ, Reddy V, Caban O. 2021. Embryology, central nervous system. In: StatPearls. Treasure Island (FL): StatPearls Publishing. [accessed 2021 Aug 25]. http://www.ncbi.nlm.nih.gov/books/NBK526024/. 30252280

[ref15] Gholipour A, Rollins CK, Velasco-Annis C, Ouaalam A, Akhondi-Asl A, Afacan O, Ortinau CM, Clancy S, Limperopoulos C, Yang E et al. A normative spatiotemporal MRI atlas of the fetal brain for automatic segmentation and analysis of early brain growth. Sci Rep. 2017:7(1):476. 10.1038/s41598-017-00525-w. 28352082 PMC5428658

[ref16] Goodlett CR, Horn KH. Mechanisms of alcohol-induced damage to the developing nervous system. Alcohol Res Health. 2001:25(3):175–184. 11810955 PMC6707174

[ref17] Götz M, Hartfuss E, Malatesta P. Radial glial cells as neuronal precursors: a new perspective on the correlation of morphology and lineage restriction in the developing cerebral cortex of mice. Brain Res Bull. 2002:57(6):777–788. 10.1016/s0361-9230(01)00777-8. 12031274

[ref18] Gupta KK, Gupta VK, Shirasaka T. An update on fetal alcohol syndrome-pathogenesis, risks, and treatment. Alcohol Clin Exp Res. 2016:40(8):1594–1602. 10.1111/acer.13135. 27375266

[ref19] Innocenti GM . Growth and reshaping of axons in the establishment of visual callosal connections. Science. 1981:212(4496):824–827. 10.1126/science.7221566. 7221566

[ref20] Jacobson SW, Jacobson JL, Molteno CD, Warton CMR, Wintermark P, Hoyme HE, De Jong G, Taylor P, Warton F, Lindinger NM et al. Heavy prenatal alcohol exposure is related to smaller corpus callosum in newborn MRI scans. Alcohol Clin Exp Res. 2017:41(5):965–975. 10.1111/acer.13363. 28247416 PMC5404976

[ref21] LaMantia AS, Rakic P. Axon overproduction and elimination in the corpus callosum of the developing rhesus monkey. J Neurosci. 1990:10(7):2156–2175. 10.1523/JNEUROSCI.10-07-02156.1990. 2376772 PMC6570389

[ref22] Lange S, Probst C, Gmel G, Rehm J, Burd L, Popova S. Global prevalence of fetal alcohol spectrum disorder among children and youth: a systematic review and meta-analysis. JAMA Pediatr. 2017:171(10):948–956. 10.1001/jamapediatrics.2017.1919. 28828483 PMC5710622

[ref23] Lebel C, Roussotte F, Sowell ER. Imaging the impact of prenatal alcohol exposure on the structure of the developing human brain. Neuropsychol Rev. 2011:21(2):102–118. 10.1007/s11065-011-9163-0. 21369875 PMC3098972

[ref24] Li Y, Zhang L-N, Chong L, Liu Y, Xi F-Y, Zhang H, Duan X-L. Prenatal ethanol exposure impairs the formation of radial glial fibers and promotes the transformation of GFAPδ-positive radial glial cells into astrocytes. Mol Med Rep. 2021:23(4):274. 10.3892/mmr.2021.11913. 33576465 PMC7893684

[ref25] Luo L, O’Leary DDM. Axon retraction and degeneration in development and disease. Annu Rev Neurosci. 2005:28(1):127–156. 10.1146/annurev.neuro.28.061604.135632. 16022592

[ref26] Mathews E, Dewees K, Diaz D, Favero C. White matter abnormalities in fetal alcohol spectrum disorders: focus on axon growth and guidance. Exp Biol Med (Maywood). 2021:246(7):812–821. 10.1177/1535370220980398. 33423552 PMC8719025

[ref27] Miller MW, Robertson S. Prenatal exposure to ethanol alters the postnatal development and transformation of radial glia to astrocytes in the cortex. J Comp Neurol. 1993:337(2):253–266. 10.1002/cne.903370206. 8276999

[ref28] Norman AL, Crocker N, Mattson SN, Riley EP. Neuroimaging and fetal alcohol spectrum disorders. Dev Disabil Res Rev. 2009:15(3):209–217. 10.1002/ddrr.72. 19731391 PMC3442778

[ref29] Nowakowski TJ, Pollen AA, Sandoval-Espinosa C, Kriegstein AR. Transformation of the radial glia scaffold demarcates two stages of human cerebral cortex development. Neuron. 2016:91(6):1219–1227. 10.1016/j.neuron.2016.09.005. 27657449 PMC5087333

[ref30] O’Leary DD, Stanfield BB, Cowan WM. Evidence that the early postnatal restriction of the cells of origin of the callosal projection is due to the elimination of axonal collaterals rather than to the death of neurons. Brain Res. 1981:227(4):607–617. 10.1016/0165-3806(81)90012-2. 7260661

[ref31] Popova S, Lange S, Probst C, Gmel G, Rehm J. Estimation of national, regional, and global prevalence of alcohol use during pregnancy and fetal alcohol syndrome: a systematic review and meta-analysis. Lancet Glob Health. 2017:5(3):e290–e299. 10.1016/S2214-109X(17)30021-9. 28089487

[ref32] Prayer D, Malinger G, Brugger PC, Cassady C, De Catte L, De Keersmaecker B, Fernandes GL, Glanc P, Gonçalves LF, Gruber GM et al. ISUOG practice guidelines: performance of fetal magnetic resonance imaging. Ultrasound Obstet Gynecol. 2017:49(5):671–680. 10.1002/uog.17412. 28386907

[ref33] Rakic P, Yakovlev PI. Development of the corpus callosum and cavum septi in man. J Comp Neurol. 1968:132(1):45–72. 10.1002/cne.901320103. 5293999

[ref34] Ramanathan R, Wilkemeyer MF, Mittal B, Perides G, Charness ME. Alcohol inhibits cell-cell adhesion mediated by human L1. J Cell Biol. 1996:133(2):381–390. 10.1083/jcb.133.2.381. 8609170 PMC2120806

[ref35] Richards LJ, Plachez C, Ren T. Mechanisms regulating the development of the corpus callosum and its agenesis in mouse and human. Clin Genet. 2004:66(4):276–289. 10.1111/j.1399-0004.2004.00354.x. 15355427

[ref36] Roediger DJ, Krueger AM, de Water E, Mueller BA, Boys CA, Hendrickson TJ, Schumacher MJ, Mattson SN, Jones KL, Lim KO et al. Hippocampal subfield abnormalities and memory functioning in children with fetal alcohol Spectrum disorders. Neurotoxicol Teratol. 2021:83:106944. 10.1016/j.ntt.2020.106944. 33232797 PMC7855420

[ref37] Sarnat HB . Embryology and malformations of the forebrain commissures. Handb Clin Neurol. 2008:87:67–87. 10.1016/S0072-9752(07)87005-9. 18809019

[ref38] Schwartz E, Diogo MC, Glatter S, Seidl R, Brugger PC, Gruber GM, Kiss H, Nenning K-H, IRC5 consortium, Langs G et al. The prenatal morphomechanic impact of agenesis of the corpus callosum on human brain structure and asymmetry. Cereb Cortex. 2021:31(9):4024–4037. 10.1093/cercor/bhab066. 33872347

[ref39] Shinar S, Har-Toov J, Lerman-Sagie T, Malinger G. Thick corpus callosum in the second trimester can be transient and is of uncertain significance. Ultrasound Obstet Gynecol. 2016:48(4):452–457. 10.1002/uog.15678. 26282069

[ref40] Shohayeb B, Muzar Z, Cooper HM. Conservation of neural progenitor identity and the emergence of neocortical neuronal diversity. Semin Cell Dev Biol. 2021:118:4–13. 10.1016/j.semcdb.2021.05.024. 34083116

[ref41] Shulman HB, D’Angelo DV, Harrison L, Smith RA, Warner L. The pregnancy risk assessment monitoring system (PRAMS): overview of design and methodology. Am J Public Health. 2018:108(10):1305–1313. 10.2105/AJPH.2018.304563. 30138070 PMC6137777

[ref42] Sokol RJ, Martier SS, Ager JW. The T-ACE questions: practical prenatal detection of risk-drinking. Am J Obstet Gynecol. 1989:160(4):863–868 discussion 868-870. 10.1016/0002-9378(89)90302-5. 2712118

[ref43] Stanfield BB . The development of the corticospinal projection. Prog Neurobiol. 1992:38(2):169–202. 10.1016/0301-0082(92)90039-h. 1546163

[ref44] Vasung L, Rollins CK, Velasco-Annis C, Yun HJ, Zhang J, Warfield SK, Feldman HA, Gholipour A, Grant PE. Spatiotemporal differences in the regional cortical plate and subplate volume growth during fetal development. Cereb Cortex. 2020:30(8):4438–4453. 10.1093/cercor/bhaa033. 32147720 PMC7325717

[ref45] Wang H, Suh JW, Das SR, Pluta JB, Craige C, Yushkevich PA. Multi-atlas segmentation with joint label fusion. IEEE Trans Pattern Anal Mach Intell. 2013:35(3):611–623. 10.1109/TPAMI.2012.143. 22732662 PMC3864549

[ref46] Warton FL, Meintjes EM, Warton CMR, Molteno CD, Lindinger NM, Carter RC, Zöllei L, Wintermark P, Jacobson JL, van der Kouwe A et al. Prenatal methamphetamine exposure is associated with reduced subcortical volumes in neonates. Neurotoxicol Teratol. 2018:65:51–59. 10.1016/j.ntt.2017.10.005. 29069607 PMC5803390

[ref47] World Medical Association . World medical association declaration of Helsinki: ethical principles for medical research involving human subjects. JAMA. 2013:310(20):2191–2194. 10.1001/jama.2013.281053. 24141714

[ref48] Yushkevich PA, Piven J, Hazlett HC, Smith RG, Ho S, Gee JC, Gerig G. User-guided 3D active contour segmentation of anatomical structures: significantly improved efficiency and reliability. NeuroImage. 2006:31(3):1116–1128. 10.1016/j.neuroimage.2006.01.015. 16545965

[ref49] Žunić Išasegi I, Milan R, Krsnik Ž, Marko R, Benjak V, Kostović I. Interactive histogenesis of axonal strata and proliferative zones in the human fetal cerebral wall. Brain Struct Funct. 2018:223(9):3919–3943. 10.1007/s00429-018-1721-2. 30094607 PMC6267252

