# Supplementary material for: Fetal MRI based brain atlas analysis detects initial in utero effects of prenatal alcohol exposure
Source: Cereb Cortex. 2023 Feb 17;33(11):6852–61. doi: 10.1093/cercor/bhad005 (PMC10233241; doi:10.1093/cercor/bhad005)
Supplement: Supplementary_material_bhad005 [file supplementary_material_bhad005.docx]

# Supplementary material

**Supplementary table 1: Indications for/diagnosis following fetal MRI**

Abbreviations: GA – gestational age, PAE – prenatal alcohol exposure, m – male, f – female

| **PAE** | **Sex** | **Reasons for referral/ suspected diagnosis** | **Final diagnosis following MRI** |
| --- | --- | --- | --- |
| + | m | corpus callosum agenesis | asymmetrical mild ventriculomegaly (7/12mm), normal corpus callosum |
| + | m | mild ventriculomegaly | mild ventriculomegaly (9/11mm) |
| + | m | lymphangioma or teratoma involving left leg | lymphangioma involving left leg |
| + | f | alcohol abuse | small head biometry |
| + | m | partial corpus callosum agenesis | no pathology |
| + | m | cleft lip and palate | no pathology |
| + | m | mild ventriculomegaly | asymmetrical ventriculomegaly (8/11mm) |
| + | f | right aortic arch | right aortic arch |
| + | f | suspected lung hypoplasia | diaphragmal eventration, lung hypoplasia |
| + | m | suspected DiGeorge | right-sided aortic arch, upper lobe bronchial compression |
| + | m | gastroschisis | gastroschisis |
| + | m | abdominal cyst | no pathology |
| + | m | subcapsular liver hematoma, rhesus incompatibility | signal alterations in liver following transfusion, rhesus incompatibility |
| + | m | ventriculomegaly | asymmetrical ventriculomegaly (12/8mm) |
| + | m | lissencephaly in previous pregnancy | no pathology |
| + | f | recent maternal COVID-19 infection | no pathology |
| + | f | alcohol abuse | small head biometry |
| + | m | club feet, genetic testing showed no pathology | club feet, genetic testing showed no pathology |
| + | m | exophthalmus | midface hypoplasia, exopthalmus |
| + | m | lymphangioma or teratoma involving left leg | lymphangioma or teratoma involving left leg |
| + | f | ovarian cyst | ovarian cyst |
| + | m | mild polyhydramnion | mild polyhydramnion, no pathology |
| + | f | plexus cysts | small bilat. plexus cysts, no additional fetal pathologies |
| + | m | cleft lip and palate | cleft right lip and palate |
| + | m | aortic isthmus stenosis | aortic isthmus stenosis |
| + | m | aortic isthmus stenosis | aortic isthmus stenosis |

| **PAE** | **Sex** | **Reasons for referral/ suspected diagnosis** | **Final diagnosis following MRI** |
| --- | --- | --- | --- |
| - | f | 22q13 deletion in previous pregnancy | no pathology |
| - | m | heterotaxy in previous pregnancy | no pathology |
| - | m | jejunal atresia | jejunal atresia |
| - | m | two cysts cranial of left kidney; suspected duodenal atresia | cyst cranial of left kidney |
| - | m | polycystic dysplastic kidney left | polycystic dysplastic left kidney |
| - | f | unilateral kidney agenesis | caudalized left kidney |
| - | m | hemorrhage in cisterna magna | no pathology |
| - | f | kidney cyst | ureterocele left |
| - | m | herniation into single umbilical artery | herniation into single umbilical artery |
| - | m | abnormal foot positioning | no pathology |
| - | m | borderline unilateral ventriculomegaly | no pathology |
| - | m | cleft lip and palate | cleft right lip and palate |
| - | m | premature rupture of membranes | premature rupture of membranes, single umbilical artery |
| - | m | unilateral kidney agenesis | unilateral kidney agenesis |
| - | m | ovarian cyst | elongated gallbladder |
| - | f | unilateral kidney agenesis | unilateral pelvic kidney |
| - | m | premature rupture of membranes | premature rupture of membranes, no fetal pathology |
| - | m | abdominal cyst | elongated gallbladder |
| - | m | VACTERL in previous pregnancy | no pathology |
| - | f | distended bowel loop | no pathology |
| - | m | renal fusion | unilateral dysplastic kidney |
| - | m | premature rupture of membranes | premature rupture of membranes, no fetal pathology |
| - | f | agenesis of left hand | agenesis of left hand |
| - | m | atresia of bile ducts | dilated gallbladder, persistent right umbilical vein |
| - | f | splenic cyst | splenic cyst |
| - | m | premature rupture of membranes | premature rupture of membranes, lung hypoplasia |
| - | f | cyst involving ductus choledochus | gallbladder agenesis |
| - | f | premature rupture of membranes | premature rupture of membranes, no fetal pathology |
| - | f | lung anomaly | subpulmonal sequester left |
| - | m | increased fetal head biometry | no pathology |
| - | f | premature rupture of membranes | premature rupture of membranes, no fetal pathology |
| - | m | borderline ventriculomegaly, macroglossia | no pathology |
| **PAE** | **Sex** | **Reasons for referral/ suspected diagnosis** | **Final diagnosis following MRI** |
| - | m | mild polyhydramnion | mild polyhydramnion |
| - | m | echodense spots in fetal liver | no pathology |
| - | m | abdominal cyst | no pathology |
| - | m | congenital cystic adenomatoid malformation | congenital cystic adenomatoid malformation |
| - | f | microcephaly | no pathology |
| - | m | premature rupture of membranes | premature rupture of membranes, no fetal pathology |
| - | m | congenital cystic adenomatoid malformation | congenital cystic adenomatoid malformation |
| - | m | connective tissue disease in previous pregnancy | no pathology |
| - | m | unilateral multicystic kidney | posterior urethral valves, multicystic kidneys |
| - | m | bilateral pelvic kidney | bilateral pelvic kidney |
| - | m | corpus callosum hypoplasia | no pathology |
| - | m | ileum stenosis, microcolon | jejunal atresia, microcolon |
| - | m | mega cisterna magna | no pathology |
| - | f | gallbladder anomaly | esophageal atresia |
| - | f | congenital cystic adenomatoid malformation | congenital cystic adenomatoid malformation |
| - | m | hypokinesia in lower extremities | no pathology |
| - | f | ovarian cyst | ovarian cyst |
| - | f | club feet, genetic testing showed no pathology | club feet, genetic testing showed no pathology |
| - | f | ovarian cyst | ovarian cyst |
| - | f | ovarian cyst | ovarian cyst |
